# Small Molecule Docking Supports Broad and Narrow Spectrum Potential for the Inhibition of the Novel Antibiotic Target Bacterial Pth1

**DOI:** 10.3390/antibiotics5020016

**Published:** 2016-05-10

**Authors:** Paul P. Ferguson, W. Blake Holloway, William N. Setzer, Hana McFeeters, Robert L. McFeeters

**Affiliations:** Department of Chemistry, University of Alabama in Huntsville, 301 Sparkman Drive, Huntsville, AL 35899, USA; ppf0001@uah.edu (P.P.F.); beb0004@uah.edu (W.B.H.); setzerw@uah.edu (W.N.S.); hk0003@uah.edu (H.M.)

**Keywords:** peptidyl-tRNA hydrolase, molecular docking, novel antibiotic target, antibiotics, enzyme inhibitors, tRNA, protein biosynthesis inhibitor

## Abstract

Peptidyl-tRNA hydrolases (Pths) play ancillary yet essential roles in protein biosynthesis by recycling peptidyl-tRNA. In *E. coli*, inhibition of bacterial Pth1 leads to accumulation of peptidyl-tRNA, depletion of aminoacyl-tRNA, and cell death. Eukaryotes have multiple Pths and Pth1 knock out was shown to have no effect on viability in yeast. Thereby, bacterial Pth1 is a promising target for novel antibiotic development. With the abundance of Pth1 structural data, molecular docking was used for virtual screening of existing, commercially available antibiotics to map potential interactions with Pth enzymes. Overall, 83 compounds were docked to eight different bacterial Pth1 and three different Pth2 structures. A variety of compounds demonstrated favorable docking with Pths. Whereas, some compounds interacted favorably with all Pths (potential broad spectrum inhibition), more selective interactions were observed for Pth1 or Pth2 and even specificity for individual Pth1s. While the correlation between computational docking and experimentation still remains unknown, these findings support broad spectrum inhibition, but also point to the possibility of narrow spectrum Pth1 inhibition. Also suggested is that Pth1 can be distinguished from Pth2 by small molecule inhibitors. The findings support continued development of Pth1 as an antibiotic target.

## 1. Introduction

Protein biosynthesis involves ribosomal translation of mRNA, during which amino acids are added to the nascent peptide chain until the ribosome reaches a stop codon. After binding of the appropriate termination factors, the ribosomal complex disassociates and releases the newly formed protein, original mRNA transcript, and associated tRNA. Approximately 10% of the time ribosomes abort translation prematurely [1] contributing to production of peptidyl-tRNA. Additionally, accumulation of peptidyl-tRNAs results from the expression of minigenes or short open reading frames [2,3,4]. Removal of the peptide moiety from peptidyl-tRNA to return limited tRNA for use in protein biosynthesis is essential for cell viability in all domains of life.

To avoid excessive build-up of peptidyl-tRNAs and resulting tRNA starvation, it is vital for cells to maintain peptidyl-tRNA hydrolase (Pth) activity [5]. Two distinct classes of Pth enzymes are known, with Pth1 originally found in bacteria and Pth2 found predominantly in archaea and eukaryotes. There is no significant sequence or structural homology between Pth1 and Pth2 enzymes and it was demonstrated that their cleavage products are different. In rabbit reticulocytes, peptidyl-AMP and tRNA-CC are the products from the enzyme reaction [6] rather than peptide and tRNA. While select bacterial species have acquired a Pth2 gene from lateral gene transfer [7], most possess a single Pth1. Archaea recycle peptidyl-tRNA via a different class of Pth, Pth2. Eukaryotes possess multiple Pths including orthologs of bacterial Pth1, archaeal Pth2, and a variety of Pth domain containing proteins. From functional studies it was shown that deletion of Pth1, Pth2 or both did not affect viability in *Saccharomyces cerevisiae* [8,9]. In *Escherichia coli*, however, Pth1 activity is essential for survival [10]. Thus Pth1 is a viable new antibiotic target, either as a stand-alone or part of a combinatorial therapy.

Furthering promise, interrupting protein biosynthesis is an antibiotic strategy proven to be effective. Moreover, it was estimated that there are between 10- and 100-fold fewer Pth1 enzymes in the typical bacterial cell than ribosomes [2]. Thus there is a significant stoichiometric advantage in targeting Pth1 over ribosomes. Interrupting protein biosynthesis at a novel target (*i.e.*, other than the ribosome) may lead to synergy when combined with many existing antibiotics.

**Figure 1:**
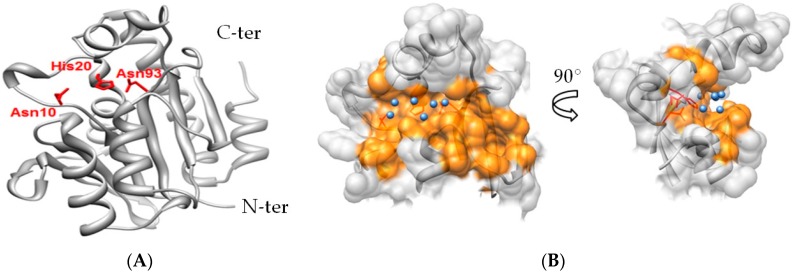
X-ray Crystal Structure of Bacterial Pth1 from *P. aeruginosa* (4FYJ). (**A**) Secondary structure of Pth1 with focus on key catalytic residues in the binding cavity (red); (**B**) Three-dimensional rendering of the peptide binding channel of Pth1, with water molecules found in the x-ray structure identified (blue).

Advancing development of Pth1 as an antibiotic target is the extensive and growing knowledge of Pth1 structural data. The crystal structure for monomeric *E. coli* Pth1 has been solved to a resolution of 1.2 Å [11]. More recently the crystal structures for Pth1 have been reported for multiple pathogenic bacterial species including *Mycobacterium tuberculosis* [12,13], *Pseudomonas aeruginosa* [14], *Francisella tularensis* [15], *Mycobacterium smegmatis* [16], *Acinetobacter baumannii* [17], *Salmonella typhimurium* [18], and *Staphylococcus aureus* [19] (coordinates yet to be released). As expected from the high degree of sequence conservation (See Supplementary Figure S1), all have related backbone folds. Pth1 family members are globular, single domain proteins composed of a central mixed β-sheet surrounded by α-helices. Three residues, Asn10, His20, and Asp93 (as numbered in *E. coli* Pth1 throughout) have been identified through site directed mutagenesis to be crucial for enzyme activity [20] (Figure 1). His20 has been speculated to act as a catalytic base in the enzymatic mechanism [21]. Despite the high degree of overall sequence similarity, significant differences are noted for residues forming the Pth1 substrate binding site that may impact small molecule binding. Insight into substrate binding and recognition comes from studies of mini-substrates [22,23], a crystal structure of Pth1 in complex with a tRNA TΨC loop [24], and a small angle scattering structure of a catalytically inactive *E. coli* Pth1 mutant bound to peptidyl-tRNA [5].

While small molecule inhibition of Pth enzymes is in its infancy, a variety of natural product extracts have been identified to inhibit bacterial Pth1s [25,26]. Differences in terms of inhibition have been observed for these natural product extracts against different Pth1s [27]. While efforts to identify individual inhibitory compounds are ongoing, computational docking can take advantage of the large amount of structural data to provide potential insight into small molecule binding.

Herein a variety of antibiotic compounds were docked to known Pth1 and Pth2 structures. Compounds already used in the clinics were chosen as a starting point since they have favorable absorption, distribution, metabolism, and excretion properties. Docking results indicate that small molecules can differentiate Pth1 from Pth2 and that narrow or even species specific Pth1 inhibition may be possible. Also found were several common interactions for highly favorable binding, considerations for future inhibitor development. Overall, these finding underscore the need for continued development of Pth1 as a novel antibiotic target and in particular, identification of small molecule inhibitors.

## 2. Results and Discussion

### 2.1. Docking Results

**Figure 2:**
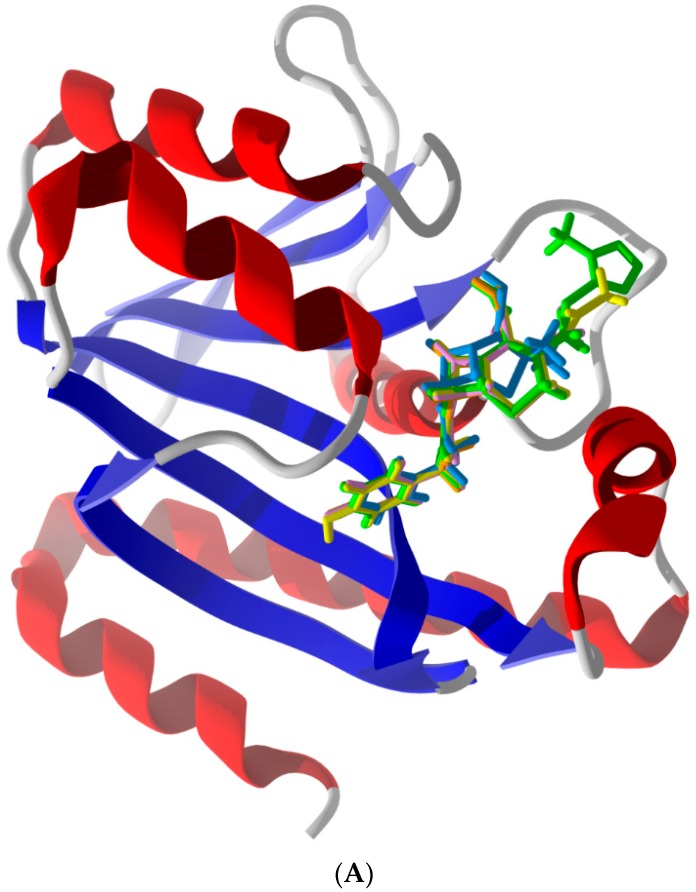

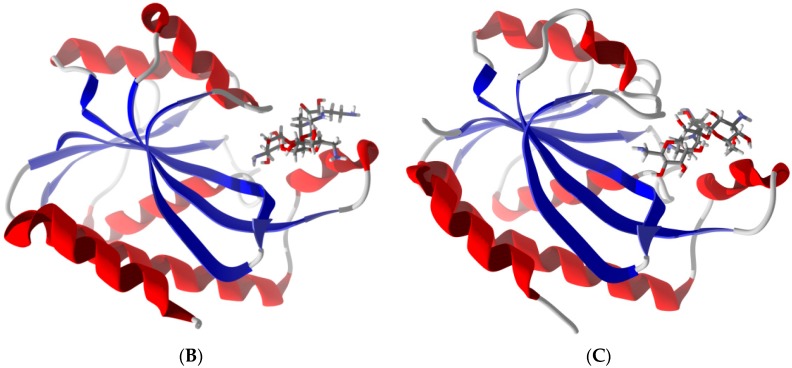
(**A**) Lowest-energy docked poses of phenylacetamide-substituted β-lactam antibiotics with *E. coli* Pth1 (PDB 2PTH): Ampicillin (aqua), cefaclor (orange), cefadroxil (pink), cefamandole (green), cefprozil (yellow), and cephalexin (lavender). The benzyl moieties of the docked ligands are sandwiched between His113 and Leu95 of the protein binding site. (**B**) Lowest-energy docked poses of amikacin with *E. coli* Pth1 (PDB 2PTH) and with (**C**) *A. baumanii* Pth1 (PDB 4FOP).

Table 1 displays the binding results determined by re-rank score for each of the Pth1s docked to the ligand set. The docking cleft was selected based on the presence of catalytically essential (His20) and catalytically important (Asn10/Asp93) residues [20]. For reference Pth1s are ordered based on lowest average ∆E_dock_ to *E. coli* Pth1 and Pth2s to *Homo sapiens* Pth2, with ∆E_dock_ defined as the absolute value of the difference between docking energies of the reference Pth and another Pth (*i.e.*, |E_RefPth_ − E_Pth_|). *S.* *typhimurium* Pth1 has the closest docking energies to *E. coli* Pth1 whereas *A. baumannii* Pth1 is most different. This trend mostly agrees with overall sequence identity. *S. typhimurium* has the highest sequence identity to *E. coli* Pth1 (93%) and both *Mycobacterium* Pth1s (~40%) and *A. baumannii* Pth1 are least identical (54% identity). Commonality in docking modes for similar ligands is shown (Figure 2A) as is the distinction for a ligand with very different energies against different Pth1s (Figure 2B,C).

### 2.2. Common Favorable Pth1 Binders

**Figure 3:**
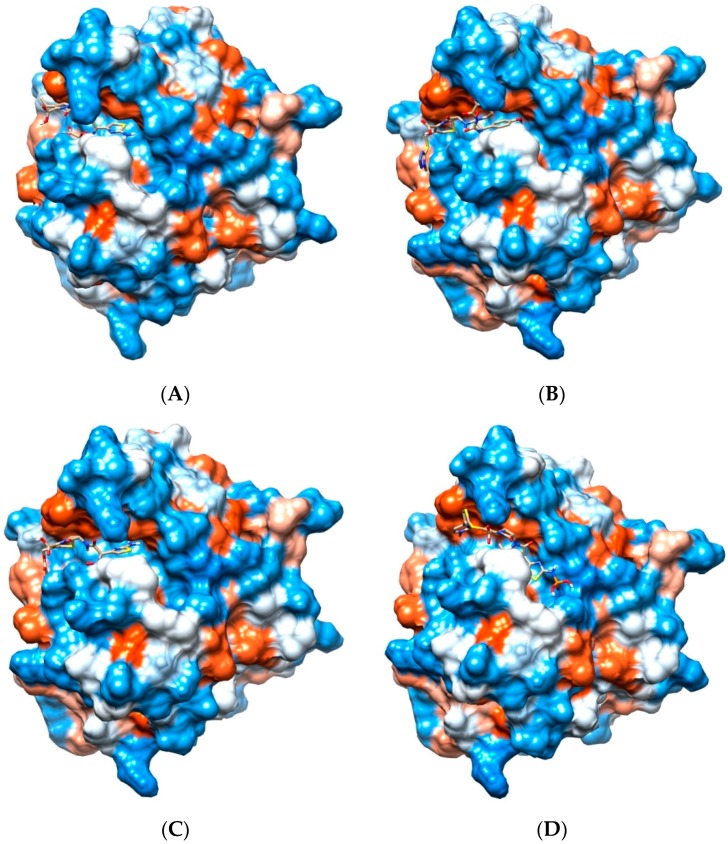
Cephalosporins and Pth1. Depicted is the hydrophobic surface of *E. coli* Pth1 (PDB 2PTH) with lowest-energy docked poses of select Cephalosporins. Polar regions are in blue, hydrophobic in red. In the binding cavity are the lowest energy conformations of (**A**) Cefixime, (**B**) Cefoperazone, (**C**) Cefotaxime, and (**D**) Ceftaroline fosamil.

Supporting previously postulated broad spectrum inhibition, four ligands demonstrated highly favorable re-rank scores (less than −90 kJ/mol) with at least seven of the eight bacterial Pth1 structures. Cefixime demonstrated highly favorable interactions with all Pth1s. Similarly, Cefoperazone, Cefotaxime and Ceftaroline fosamil also showed high favorable interactions with seven of eight docked Pth1 interactions. The weakest re-rank scores for Cefotaxime and Ceftaroline fosamil were for *A. baumanii* Pth1 at −87.6 and −82.3 kJ/mol, respectively. Cefoperazone showed some discrimination against *M. smegmatis* Pth1 (−79.6 kJ/mol), 12.4 kJ/mol below the next lowest re-rank score (*B. thailandensis* Pth1, −92.0 kJ/mol) and 43.6 kJ/mole less than the most favorable re-rank score (*E. coli* Pth1, −123.2 kJ/mol). Figure 3 shows these four ligands interacting with *E. coli* Pth1.

All of these compounds are Cephalosporins, a subgroup of β-lactam antibiotics. While the specifics of each interaction vary slightly, there are notable consistencies. Each has aromatic rings attached to the central β-lactam core that dock in hydrophobic regions of the Pth1 substrate binding cavity. In addition to the hydrophobic interactions, hydrogen bonds occur between the ligands and amino acid sidechains found in the binding cavity. Cefixime forms hydrogen bonds with Asn68 and Asn114; Cefoperazone with Leu95, Asp93, Gly112, Asn114, and Asn68; Cefotaxime with Asn68, Asn114, His20, and Asp93; and Ceftaroline fosamil with Lys142. While the most common interaction with Asn114 and almost entirely hydrophobic interaction of Ceftaroline fosamile are of particular interest, since these compounds have favorable re-rank scores with Pth2 enzymes as well, it suggests they mimic core interactions likely related to substrate recognition. In particular, the Cephalosporin core ring structure (Figure 3) may potentially mimic a nucleic acid base in terms of interacting with Pth1. While purely speculative, this would agree with the report that uridine tightly binds Pth1 in the active site [17].

### 2.3. Notable Hydrogen Bonding

Beyond compounds that were common to a majority of Pth1s, analysis of ligand docking revealed several common hydrogen bonding partners. Asp93, one of the conserved and catalytically important residues, was the most common hydrogen bonding partners for all the ligands docked with nearly one quarter of the compounds having Hydrogen bonds with it (~25% of compounds have Hydrogen bonds). The other conserved catalytic residues were far less prominent in terms of hydrogen bond formation (<5%). Other residues with notable hydrogen bonding were those at positions equivalent to *E. coli* Pth1 Gly111 and Asn114 (~15% for each) and to a lesser extent Glu94 and Leu95 (~10% for each).

### 2.4. Potential for Pth1 Ligand Selectivity

From an overall perspective, docked ligands that interacted strongly with Pths could generally be placed into the following major categories: those having favorable interactions with (1) individual Pth1s, (2) multiple Pth1s, (3) all but a single Pth1 (selective exclusion), and (4) those exhibiting Pth2 preference over Pth1. While there seems to be considerable diversity in small molecule docking of the tested compounds, several ligands stand out for their ability to potentially selectively interact with individual or subsets of Pth1 species. Compounds in category 1 that showed high selectivity (∆E > ~20 kJ/mol) for an individual Pth1 were Cefdinir, Enoxacin, Fosfomycin, Mafenide, and Streptomycin, interacting favorably with *A. baumannii* Pth1. Cefdinir was the only one that did not dock with any of the other Pth1s. Gentamicin C, Nalidixic Acid, Sulfisoxaxole, and Sulfasalazine docked preferably to *E. coli* Pth1. Streptomycin favored *B. thailandensis* Pth1 whereas Sulfisoxaxole favored *S. typhimurium* Pth1. Compounds in the second category, those that showed highly favorable interactions to multiple Pths, were Herbimycin, Imipenem, Metronidazole, and Netilmicin, all with preference for *E. coli* and *A. baumannii* Pth1s, and Lomefloxacin with preference for *E. coli* and *S. typhimurium* Pth1s. Cefadroxil strongly interacted with *E. coli* and *A. baumannii* Pth1s. Sulfasalazine seemed to display some degree of selectivity, demonstrating highly favorable interactions with *E. coli*, *S. typhimurium*, *B. thailandensis*, and *P. aeruginosa* Pth1s (but not *F. tularensis* Pth1). Two compounds in this category were very similar to those in category 3, having one interaction very near the highly favorable cutoff of −90 kJ/mol. Ertapenem demonstrated highly favorable interactions with all Pth1s except *M. smegmatis* (−88.3 kJ/mol) and *A. baumannii* Pth1. Similarly Mezlocillin demonstrated highly favorable interaction with all Pth1s except *A. baumannii Pth1* (−88.1 kJ/mol) and *E. coli* Pth1, respectively.

Multiple compounds showed less favorable re-rank scores for one Pth1, representing category 3. Amikacin, Azlocillin, Aztreonam, Cefazolin, Ceftizoxime, (Ceftibuten weaker), Ciprofloxacin, Penicillin G, Sulfacetamide, Tigecycline docked more weakly with *A. baumanii* Pth1 compared to all the other Pth1s. Similar is true for Carbeniciliin which does not dock with *A. baumannii* Pth1, but also has considerably weaker binding to *F. tularensis* Pth1. Other compounds that display weaker binding to select Pth1s also included Penicillin V and Puromycin weakly interacting with *E. coli* Pth1; Cefamandole and Cloxacillin weakly interacting with *B. thailandensis* Pth1; and Cefoxitin and Tinidazole weakly interacting with *P. aeruginosa* Pth1. Anisomycin showed considerably weaker interactions for both *Mycobacterium* species. Overall it appears that small molecules can differentiate between Pth1s supporting the possibility of both broad and narrow spectrum Pth1 inhibition.

### 2.5. Other Compounds of Note

Several compounds that do not fit well into the previously defined categories are of interest, in particular for their impact on establishing structure activity relationships. Chloramphenicol demonstrated interactions directly related to sequence identity, strongly interacting with *E. coli*, *S. typhimurium*, and *B. thailandensis* Pth1s, moderately with *F. tularensis* Pth1, and mostly weakly with *P. aeruginosa*, both *Mycobacterium* and *A. baumanii* Pth1s. A similar pattern was observed for Doripenem with the exception of the interaction with *A. baumannii* Pth1. Doxycycline is of interest for its strong interaction preference for with *A. baumannii* Pth1 but inability to dock to *B. thailandensis* Pth1. Countering the general likeness between the two highly sequence similar Pth1s, Kanamycin, Mezlocillin, Penicillin V, and Puromycin interact considerably more favorably with *S. typhimurium* Pth1 than *E. coli* Pth1 (>40 kJ/mol difference). Gentamicin C, Herbimycin, Oxytetracycline, Tetracycline display the opposite trend, suggesting that very similar Pth1s can be differentiated by small molecules.

Other compounds of interest include Meropenem which had highly favorable interactions with *S. typhimurium*, *B. thailandensis*, and *A. baumannii* Pth1s, but very weak (−35.6 kJ/mol) with *P. aeruginosa* Pth1. Methicillin shows highly favorable interactions with *E. coli* and *A. baumannii* Pth1s, similar to several category 2 compounds, but was not sufficiently selective in that the other Pth1s are close in binding energy. Minocycline showed favorable interactions with *E. coli* Pth1 but did not dock *B. thailandensis* Pth1. Moxifloxacin and Sulfamethizole, like Chloramphenicol, followed the general trend of sequence similarity. Ofloxacin demonstrated strong interaction with *A. baumanii* Pth1, but weak with *B. thailandensis*. Both Penicillin G and Platensimycin interacted strongly with *E. coli* and weakly with *A. baumannii* Pth1. However, Penicillin G displayed highly favorable binding with *S. typhimurium* Pth1 while Platensimycin did with *P. aeruginosa* Pth1. Ertapenem and Cefditoren displayed the most favorable interactions of all, both to *E. coli* Pth1. Cefditoren was much more promiscuous with a minimum Pth1 binding energy of −81.8 kJ/mol (*F. tularensis* Pth1) whereas Ertapenem displayed the biggest differences across Pth1s with weak binding to *A. baumannii* Pth1 (∆E = 118.8 kJ/mol to *E. coli* Pth1).

### 2.6. Differentiation of Pth1 from Pth2

In addition to differential binding amongst Pth1s, multiple compounds showed a general preference for either Pth1s or Pth2s. Moxifloxacin was prominent with an average re-rank score of −81.1 kJ/mol and average Pth2 re-rank score of −48.2 kJ/mol, though considerable variation was observed for Pth2 binding. Cloxacillin and Ertapenem also showed large differences between average Pth1 and Pth2 re-rank scores, though both had Pths that countered the trend. Of most interest for development of antibiotics, several other compounds appeared to discriminate well between Pth1s and Pth2 from *H. sapiens*. With occasional exceptions for individual Pth1s, Dicoxacillin and Tobramycin favored Pth1 binding. Conversely Cephadroxil, Cefepime, Fosfomycin, Geldanamycin, Herbimycin, Mafenide, Metronidazole, Nalidixic acid, Pennicillin G, and Tigecycline favored Pth2 from *H. sapiens* over Pth1s. Thus docking suggests small molecules can be used to distinguish Pth1 from Pth2, much more readily Pth1s *versus* a single species of Pth2 (*i.e.*, *H. sapiens* Pth2). Similarly, many more compounds distinguish a single Pth1 *versus* *H. sapiens* Pth2, adding promise to developing Pth1 as an antibiotic target.

## 3. Materials and Methods

### 3.1. Computational Methods

Protein-ligand docking simulations were conducted using Molegro Virtual Docker v. 5.0 [28,29]. Pth1 enzymes and ligands were desolvated and co-crystallized ligands were excluded from the complex structures. Molecular docking calculations for each complex occurred within a sphere large enough to accommodate the substrate binding cavity of the Pth and enough space to allow each ligand to search for possible docking conformations. Standard protonation states based on neutral pH were used throughout. Charges were assigned on each Pth based on templates included in the library files of Molegro Virtual Docker. Flexible ligand models were used in the docking and subsequent optimization. Variable orientations of each ligand were searched and ranked based on their re-rank score. For each docking simulation the maximum number of iterations for the docking algorithm was set to 1500, with a maximum population size of 50, and 100 runs per ligand. The RMSD threshold for multiple poses was set to 1.00 Å. The generated poses from each ligand were sorted by the calculated re-rank score, and those with the strongest re-rank scores are displayed in Table 1. Re-rank scores that occurred beyond two standard deviations more negative than the average score for that particular ligand were considered selective.

### 3.2. Ligand Dataset

A dataset of compounds has been assembled from common antibiotics, with compound structures shown in Supplementary Figure S2. The three-dimensional structure of each ligand was generated using Spartan’10 [30], where the lowest energy conformer was found in the ground state using the Hartree-Fock 6–31G* method and saved as a pdb file for import into Molegro Virtual Docker.

### 3.3. Protein Structure Data Set

All Pth structural coordinates were obtained from the Protein Data Bank and each structure was analyzed for docking against the antibiotic ligands. The Pth1 set included eight bacterial structures: *Escherichia coli* (PDB 2PTH) [11], *Mycobacterium smegmatis* (3KJZ) [16], *Mycobacterium tuberculosis* (2Z2I) [13], *Pseudomonas aeruginosa* (4FYJ) [14], *Francisella tularensis* (3NEA) [15], *Burkholderia thailandensis* (3V2I) [31], *Acinetobacter baumannii* (4FOP) [17], and *Salmonella typhimurium* (4P7B) [18]. Also included are Pth2 structures from *Archaeoglobus fulgidus* (3ERJ) [7] and *Sulfolobus solfataricus* (1XTY) [32] as well as Pth2 from *Homo sapiens* (1Q7S) [33].

## 4. Conclusions

While computational docking can be quite informative, the inherent limitations must be kept in mind. Coordinates from crystal structures may not reflect populated conformations in solution, especially for highly dynamic regions. This is of particular relevance for Pth1 since helix-4, central to forming the substrate binding pocket, is known to be mobile on biologically relevant time-scales [12]. Until experimental evidence is available to bridge the gap between docking and *in vitro* or *in vivo* inhibition, caution must be exercised in the interpretation of docking results. Thus the authors have placed emphasis on general observations, utilizing multiple individual examples to support general observations.

With this approach in mind, several worthwhile notable observations stand out. First, docking results suggest small molecules can distinguish and selectively bind to the substrate recognition sites of Pth1 *versus* Pth2. While the amino acid sequence of Pth1 is very different from Pth2, as are the reaction products and catalytic mechanism, both enzyme types recognize the same peptidyl-tRNA substrates. The differences in active site, in particular active site residues, lend themselves to small molecule differentiation. It thus seems possible to interrupt Pth1 without interfering with Pth2 activity, a significant though not necessarily absolute positive for continued Pth1 antibiotic development. Since loss of Pth1 activity in eukaryotic cells does not alter viability, yet Pth1 activity is essential for most bacteria, promise is indicated for further development of Pth1 as an antibiotic target.

Another notable observation from the docking results suggests that species specific discrimination of Pth1 is possible by small molecules. For several Pth1s in this study, one or more compound show a very large deviation in binding energy for a particular homolog. Thus significant differences in the interaction (attributed to amino acid substitutions in and around the binding cleft), potentially discriminable by small molecules, exist. While broad spectrum inhibition has been postulated for Pth1s, this finding indicates the possibility of narrow spectrum inhibition which is of great interest to potentially mitigate rapid acquisition of resistance.

Clearly, future experimentation will be required to ensure consistency between computational observation and *in vitro* results. Fortunately, significant advances in quantitating Pth activity have been made [34,35]. Coupled with the availability of recombinant production of multiple Pth species, that possibility is nearer to reality than ever.

## Figures and Tables

**Table 1:** MolDock docking energies (re-rank scores, kJ/mol) for antibiotic ligands.

Compound	Pth1								Pth2		
	*Eco ^1^*	*Sty*	*Bth*	*Ftu*	*Par*	*Mtb*	*Msm*	*Aba*	*Hs*	*Ss*	*Af*
	2PTH	4P7B	3V2I	3NEA	4FYJ	2Z2I	3KJZ	4FOP	1Q7S	1XTY	3ERJ
Amikacin	−80.3	−77.1	−81.1	−60.2	−82.8	−91.7	−67.5	−23.7	−90.1	−94.1	−81.1
Amoxicillin	−88.1	−87.4	−89.7	−34.8	−76.7	−75.2	−75.9	−95.3	−87.9	−82.8	−78.7
Ampicillin	−93.4	−81.9	−84.4	−81.4	−74.4	−71.2	−77.9	−86.6	−85.6	−92.6	−64.9
Anisomycin	−90.9	−95.6	−87.6	−80.7	−84.3	−63.7	−63.4	−93.7	−68.3	−69.9	−76.7
Azlocillin	−114.6	−119.7	−106.4	−85.4	−112	−101.5	−90	−52.8	−40.5	−120.2	nd
Aztreonam	−104.8	−92.2	−98.5	−105.2	−76.2	−82.6	−86.8	−23.4	−73	−91.9	−94.6
Blasticidin S	−89.5	−99	−94.5	−92.5	−80.8	−76.4	−77.6	−86.4	−82.6	−94.5	−70.2
Capreomycin	−114.3	−95.6	−75.3	−71.4	−90.6	−83.5	−79	−103.5	−90.4	−108	−81.1
Carbenicillin	−101.9	−88.5	−87.3	−26.4	−91.3	−76.7	−77.1	nd	nd	−94.6	−65.9
Cefaclor	−102.7	−84.8	−91.3	−76.3	−56.5	−71.6	−69.4	−97.7	−102.7	−84	−74.4
Cefadroxil	−102.7	−96.4	−90.6	−85.7	−84.8	−73.7	−81	−112.1	−120.8	−85.5	−73.2
Cefamandole	−110.6	−92	−38.2	−97.6	−73	−94.5	−91.6	−96.6	−95.3	−96.6	−86.5
Cefazolin	−118.3	−115.5	−93.7	−99.4	−68.5	−93.6	−104	−53.7	−97.1	−98.7	−90.8
Cefdinir	nd	nd	nd	nd	nd	nd	nd	−99.2	−90.9	nd	nd
Cefditoren	−132	−107.6	−100.4	−81.8	−108.3	−98.6	−89	−100.9	−94.7	−117.7	−89.1
Cefepime	−105.7	−99.1	−96.3	−100.4	−96.3	−85	−91.2	−87.1	−122.5	−106.9	−96.8
Cefixime	−115.2	−110	−106.8	−106.7	−98	−98.1	−94.8	−118.2	−102.4	−108.1	−96.2
Cefoperazone	−123.2	−115.3	−92	−109.8	−93.8	−99.4	−79.6	−116.8	−100.6	−101.1	−109.1
Cefotaxime	−105.8	−98.1	−103.6	−121.5	−93	−91.3	−92.2	−87.6	−89.6	−100.4	−101.7
Cefoxitin	−103.4	−88	−85.5	−105.9	−51.3	−83.3	−83.7	−90.9	−82.1	−97.2	−78.1
Cefpodoxime	−105.2	−93.7	−110	−110.3	−92.2	−86.9	−84.6	−91.7	−82.3	−95	−97.5
Cefprozil	−106.5	−90.3	−94.2	−92.8	−104.1	−83.4	−84.3	−90.4	−97.4	−85.3	−70.5
Ceftaroline fosamil	−118.6	−117.4	−112.3	−92	−96.9	−114.9	−98.6	−82.3	−81.5	−134.1	−110.5
Ceftazidime	−108.3	−94.3	−93.7	−107.9	−104.8	−83.9	−86.3	−81.4	−75.6	−110.1	−103.1
Ceftibuten	−104.8	−108.7	−97.2	−100.5	−100.8	−87.6	−89.3	−66.1	−86.6	−101.1	−103.7
Ceftizoxime	−103.9	−100.4	−101.9	−96.1	−73.3	−85.7	−86.9	−95.9	−84.1	−97.4	−90.5
Cephalexin	−103.1	−80.4	−89.4	−82.1	−85	−76.8	−80.6	−66.5	−74.9	−84.5	−81.1
Cephalothin	−111.4	−96.2	−95.8	−80.7	−103.8	−87.5	−84.8	−98	−76.9	−92.2	−87.7
Chloramphenicol	−92.1	−91.4	−91	−83.4	−63.3	−66.1	−65.3	−69.3	−68.9	−80.5	−84.9
Ciprofloxacin	−87.4	−78.4	−66.8	−98.3	−84.1	−69.7	−64.3	−46.9	−43.5	−89.7	−75.2
Clindamycin	−85.1	−86.1	−85.1	−83.5	−88.2	−83.3	−74.7	−85	−86.2	−81.4	−78.6
Clofazimine	−99.3	−75.8	−99.3	−78.7	−90.6	−90.1	−66.2	−71.5	−70.9	−74.9	−72.5
Cloxacillin	−95.8	−84.3	−47.9	−94	−97.5	−91.2	−82.5	−88	−41.6	−88.4	−25.6
Dicloxacillin	−81.6	−77.7	−85.8	−93.9	−99	−89.8	−79.8	−71.3	−50.6	−95.2	−64.6
Doripenem	−104.6	−113.8	−105.9	−107.2	−83.4	−92.4	−81	−105.4	−82.2	−94.1	−70.4
Doxycycline	−61.4	−57.9	nd	−77	−62.8	−66.5	−51.3	−103.1	−89.5	−78.4	−59.5
Enoxacin	−83.6	−74.5	−66.4	−95.1	−73.9	−64.4	−60.8	−96.4	−81.3	−84.7	−88.5
Ertapenem	−137.7	−128.9	−106.3	−111.7	−123.3	−102	−88.3	−18.9	−25	−107.2	−74.7
Flucloxacillin	−90.9	−82.8	−88.3	−93.3	−98.9	−90.4	−77.4	−83.6	−72.9	−88.6	−81.4
Fosfomycin	−43.7	−48.5	−42.7	−43.1	−43.8	−31.6	−40.7	−105.6	−85.7	−38.8	−37.5
Furazolidone	−90.6	−95.7	−84.9	−76	−82.2	−68.7	−66.6	−78.8	−76.9	−72.8	−84.3
Gatifloxacin	−59.4	−81.5	−53.4	−98.9	−89.2	−65.9	−61.8	−82.3	−78.8	−86.2	−27.8
Geldanamycin	−75.5	−60.8	−67.8	−70	−74.2	−75.4	−59.4	−62.8	−99.9	−74.6	−57.2
Gentamicin C	−109.5	−64	−86.2	−87.7	−69.4	−75.1	−53.7	−64.3	−51.3	−81.1	−67.8
Herbimycin	−91.5	−47.1	−63.7	−55.2	−58.2	−65.4	−62.8	−95.8	−91.4	−63.6	−62.9
Imipenem	−128	−105.7	−95.7	−83.7	−90.8	−80.8	−90.3	−116.5	−116	−81	−33
Kanamycin	−52.7	−102.7	−81.4	−103.1	−89.6	−83.4	−71.6	−74.4	−89.7	−99.5	−73.4
Levofloxacin	−76.2	−75.4	−79.8	−65.5	−81.1	−58.3	−58	−37.8	−78.3	−73.2	−29.9
Lincomycin	−96	−88.1	−85.8	−80.6	−83.9	−79.3	−68.9	−85.1	−86.8	−87.1	−62.2
Linezolid	−87.6	−98.9	−91.3	−79.3	−98.9	−78.6	−80.1	−77.9	−87.5	−92.5	−69.1
Lomefloxacin	−102.1	−96.6	−64.5	−73.9	−84.5	−73.2	−67.1	−78.4	−66.5	−80.5	−92.4
Loracarbef	−75	−76	−86.4	−74	−84.3	−74.3	−78.1	−86.1	−68.9	−82.2	−70.8
Mafenide	−64.1	−67.5	−58.4	−71.4	−66.3	−49.4	−52.6	−97.2	−95.6	−53.6	−56.5
Meropenem	−86.2	−106.8	−109.4	−94.5	−35.6	−86.9	−75.3	−111.4	−112.9	−91.1	−76.6
Methicillin	−92.1	−82.9	−76.1	−79.8	−76	−69.9	−67.3	−100.6	−95.2	−96.6	−47.1
Metronidazole	−87.4	−64.5	−66.2	−64.2	−66	−53.3	−48.8	−80.3	−93.1	−52.2	−48.8
Mezlocillin	−76.1	−123.3	−100.9	−102	−128.9	−91.6	−95.8	−88.1	−78.9	−97.9	−76.2
Minocycline	−94.8	−64.2	nd	−49.8	−68.4	−66.2	−63.8	−83.4	−66.1	−72.8	−91.1
Moxifloxacin	−92.2	−82.1	−85.5	−99.3	−90	−68.8	−65.3	−65.9	−47.6	−70.9	−26.2
Nalidixic acid	−111.9	−73.6	−57.6	−78.6	−68.6	−49.5	−58	−76.1	−91.1	−61.6	−78.3
Neomycin	−87	−77.4	−64.3	−67.8	−86.1	−81.8	−67.7	−77.5	−57.7	−96	−95.7
Netilmicin	−93.5	−68	−74.7	−76.4	−71.5	−80.4	−53.7	−94.9	−72.4	−77.1	−30.9
Norfloxacin	−77	−73.8	−67.8	−72.7	−54.7	−68.5	−61.9	−88.5	−85	−86	−93.2
Ofloxacin	−53	−64.3	−36.4	−75.8	−70.3	−69.9	−58.5	−102.7	−82.5	−68	−92.7
Oxytetracycline	−99.4	−51.7	−57.8	−78.4	−64.9	−57.5	−55.1	−87.4	−73.1	−81.9	−60.2
Penicillin G	−106.6	−98.5	−86	−76.1	−90.7	−73.2	−78.6	−50.4	−67.1	−92.1	−72.4
Penicillin V	−54.1	−95.5	−68.3	−83.2	−87.8	−80.2	−72.7	−81	−100	−87.7	−44.6
Platensimycin	−121.8	−93.3	−103.5	−78.1	−36.5	−79.5	−78.3	−25.3	−77.9	−85.3	−82
Puromycin	−53.8	−96.6	−109.6	−93.2	−111.5	−99.4	−81.1	−79.2	−69.1	−105.7	−89.1
Sparsomycin	−91.5	−104.1	−90.6	−89.1	−101.5	−75.9	−93.1	−79.6	−81.2	−81.5	−60.6
Streptogramin A	−93.3	−73.2	−84.5	−69.7	−78.7	−99.4	−68.9	−104.9	−102.4	−87.3	−76.4
Streptomycin	−69.8	−91.2	−97.6	−84.7	−78.6	−77.8	−74.9	−71.5	−61.1	−89.5	−63.3
Sulfacetamide	−86.1	−70.9	−66.3	−68.1	−72.7	−55.8	−52.6	−35.3	nd	−59.8	−67.5
Sulfadiazine	−93.5	−85.6	−74.4	−67.3	−73.5	−63.3	−58.1	−57.4	−77	−71.3	−74.7
Sulfamethizole	−87.5	−99.1	−80.1	−81.1	−93.4	−67.3	−67	−65.4	−64.4	−71.5	−76.4
Sulfamethoxazole	−114.8	−99.9	−80.9	−78.6	−95.5	−67.4	−72.2	−74.5	−73.2	−71	−63.7
Sulfasalazine	−95.1	−107.7	−107	−71.2	−110.9	−82.3	−75.9	−73.9	−88	−85.7	−84.7
Sulfisoxazole	−78.8	−102.4	−80.1	−77.1	−89	−71.7	−70.3	−64.2	−79.8	−75.3	−64.2
Tetracycline	−103.1	−51.1	−63.2	−78.3	−52.3	−58.2	−57.2	−88.9	−82.9	−60.6	−58.4
Thiamphenicol	−65.2	−91.1	−89.4	−85.5	−88.9	−67.3	−65.2	−75.7	−58.4	−77.7	−41.5
Tigecycline	−69.7	−63.7	−79.4	−63.7	−78.4	−89.6	−74.2	nd	−93.8	−85	nd
Tinidazole	−86	−62.3	−71.9	−76.3	−16.4	−62.4	−58.3	−81.5	−73.8	−68.6	−51.5
Tobramycin	−91.5	−79.9	−77.3	−78.4	−75.5	−77.9	−64.5	−55.8	−47.8	−95.8	−65.7

^1^ Three letter codes for Pth1 species are Eco = *E. coli*, Sty = *S. typhimurium*, Bth = *B. thailandensis*, Ftu = *F. tularensis*, Par = *P. aeruginosa*, *Mtb = M. tuberculosis*, Msm = *M. smegmatis*, Aba = *A. baumannii* whereas 2 letter codes designate Pth2s as Hs = *H. sapiens*, Ss = *S. solfataricus*, Af = *A. fulgidus*. Four character PDB accession numbers are included below each Pth species. No docking is designated “nd”.

## Supplementary Materials

The following are available online at www.mdpi.com/2079-6382/5/2/16/s1, Figure S1: Pth1 Amino Acid Sequence Conservation, Figure S2: Compound Structures.

## Abbreviations

The following abbreviations are used in this manuscript:

Pth: Peptidyl-tRNA hydrolase
Pth1: bacterial peptidyl-tRNA hydrolase
