# Supplementary material for: Small Molecule Docking Supports Broad and Narrow Spectrum Potential for the Inhibition of the Novel Antibiotic Target Bacterial Pth1
Source: Antibiotics (Basel). 2016 May 10;5(2):16. doi: 10.3390/antibiotics5020016 (PMC4929431; doi:10.3390/antibiotics5020016)
Supplement: Supplementary file 1 [file antibiotics-05-00016-s001.pdf]

Supplementary Materials: Small Molecule Docking Supports Broad and Narrow Spectrum Potential for the Inhibition of the Novel Antibiotic Target Bacterial Pth1

Paul P. Ferguson, W. Blake Holloway, William N. Setzer, Hana McFeeters and Robert L. McFeeters

```
                                    10        20        30        40        50
E. coli   (2PTH)  -------------TIKLIVGLANPGAEYAATRHNAGAWFVDLLAERLRAPLREEAKFFGYTSR
S. typi.  (4P7B)  -------------AIKLIVGLANPGAEYAATRHNAGAWYVDLLAERLRAPLREEPKFFGYTSR
P. aeru.  (4FYJ)  ------------TAVQLIVGLGNPGPEYDQTRHNAGALFVERLAHAQGVSLVADRKYFGLVGK
A. baum.  (4FOP)  -----------MSNISLIVGLGNPGSEYAQTRHNAGFWFVEQLADKYGITLKNDPKFHGISGRG
B. thai.  (3V2I)  MGTLEAQTQGPGSMIKLIVGLGNPGAEYTATRHNAGFWLVDQLAREAGATLRDERRFHGFYAK
F. tula.  (3NEA)  SSGLVPRGSH-MPKIKMIIGLGNIGKEYQDTRHNVGEWFIAKIAQDNNQSFSSNPKLNCNLAK
M. tube.  (2Z2I)  -----------MAEPLLVVGLGNPGANYARTRHNLGFVVADLLAARLGAKFKAHKRSGAEVAT
M. smeg.  (3KJZ)  -----------MAEPLLVVGLGNPGPTYAKTRHNLGFMVADVLAGRIGSAFKVHKKSGAEVVT

     60        70        80        90        100       110       120
VTLGGEDVRLLVPTTFMNLSGKAVAAMASFFRINPDEILVAHDELDLPPGVAKFKLGGGHGGHNGLKDIISKLGNN
ITLEGEDVRLLVPTTFMNLSGKAVGAMASFYRIQPDEILVAHDELDLPPGVAKFKLGGGHGGHNGLKDIISKLGNN
FSHQGKDVRLLIPTTYMNRSGQSVAALAGFFRIAPDAILVAHDELDMPPGVAKLKTGGGHGGHNGLRDIIAQLGNQ
NIEGHDVRLLLPMTYMNRSGQSVVPFSKFYQIAPEAILIAHDELDMNPGVIRLKTGGGHGGHNGLRDIVPHIGP-
ARLYGEEVHLLEPQTYMNRSGQSVVALAHFFKILPNEILVAHDELDLPPGAVKLKLGGGSGGHNGLKDISAHLSSQ
VSIDYNNVVLVFPTTYMNNSGLAVSKVANFYKIAPAEILVVHDELDIDSGEIRLKKGGGHGGHNGLRSINQHLGTN
GRSAGRSLVLAKPRCYMNESGRQIGPLAKFYSVAPANIIVIHDDLDLEFGRIRLKIGGGEGGHNGLRSVVAALGTK
GRLAGTTVVLAKPRISMNESGRQVGPLAKFYSVPPQQIVVIHDELDIDFGRIRLKLGGGEGGHNGLRSVASALGTK

130       140       150       160       170       180       190       200
PNFHRLRIGIGHP-----------GDKNKVVGFVLGKPPVSEQKLIDEAIDEAARCTEMWFTDGLTKATNRLHAFKAQ
PNFHRLRVGIGHP-----------GDKNKVVGFVLGKPPVSEQKLIDEAIDEAARCTELWFKEGLAKATSRLHTFKAQ
NSFHRLRLGIGHP-----------GHSSLVSGYVLGRAPRSEQELLDTSIDFALGVLPEMLAGDWTRAMQKLHSQK--
-NFHRLRIGIGHP-----------GSKERVSGHVLGKAPSNEQSLMDGAIDHALSKVKLLVQGQVPQAMNQINAYKPA
-QYWRLRIGIGHPRDMIPESARAGAKPDVANFVLKPPRKEEQDVIDAAIERALAVMPAVVKGETERAMMQLHRNGA-
-DYLRLRIGIGHP-----------GHKSKVANYVLSNPSIAQKKDIDSAIDNGICFLDDIINYKLEPVMQKL------
-DFQRVRIGIGRP-----------PGRKDPAAFVLENFTPAERAEVPTICEQAADATELLIEQGMEPAQNRVHAW---
-NFHRVRIGVGRP-----------PGRKDPAAFVLENFTSAERAEVPTIVEQAADATELLIAQGLEPAQNTVHAW---
```

Figure S1. Amino Acid Sequence Alignment of Pth1s. Number corresponds to *E. coli* Pth1.

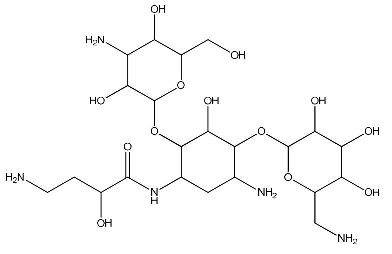

Amikacin

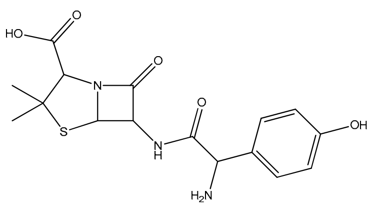

Amoxicillin

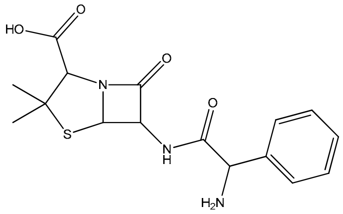

Ampicillin

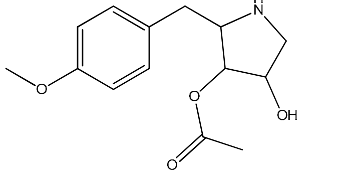

Anisomycin

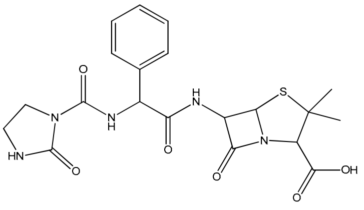

Azlocillin

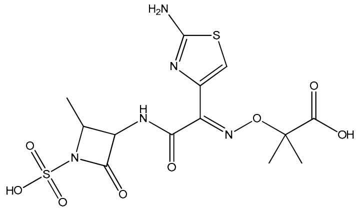

Aztreonam

Figure S2. *Cont.*

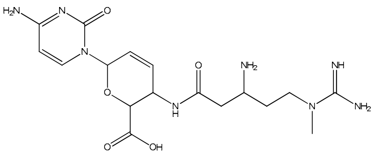

Blasticidin S

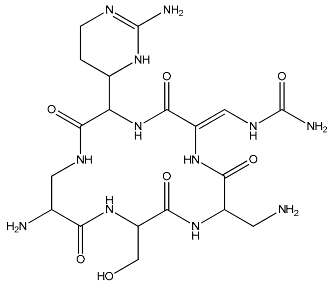

Capreomycin

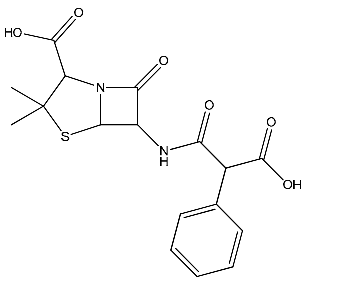

Carbenicillin

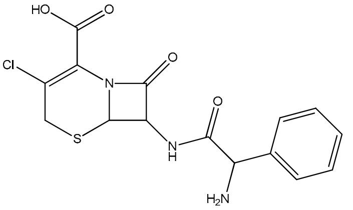

Cefaclor

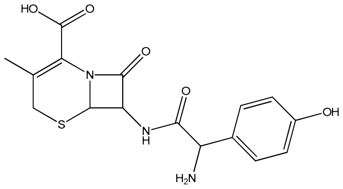

Cefadroxil

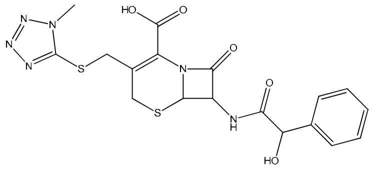

Cefamandole

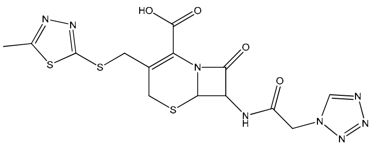

Cefazolin

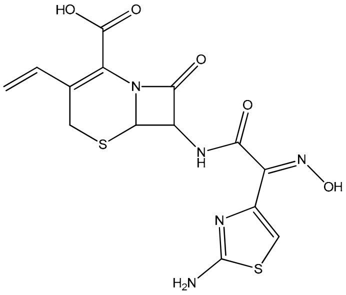

Cefdinir

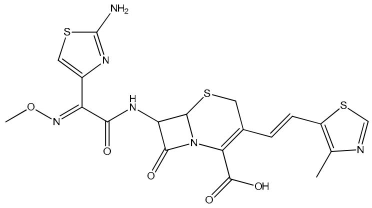

Cefditoren

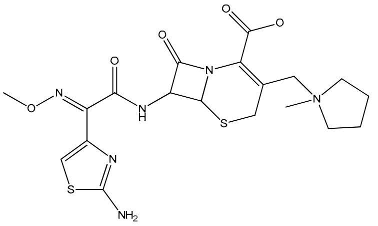

Cefepime

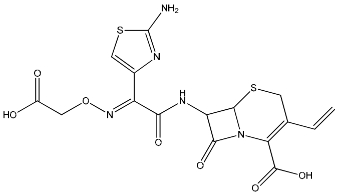

Cefixime

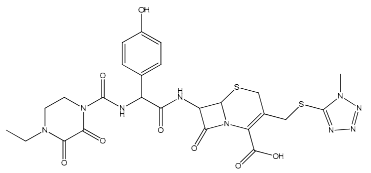

Cefoperazone

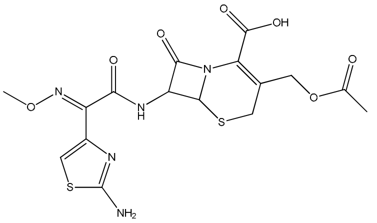

Cefotaxime

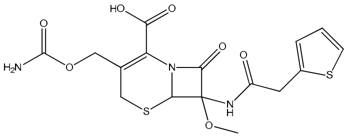

Cefoxitin

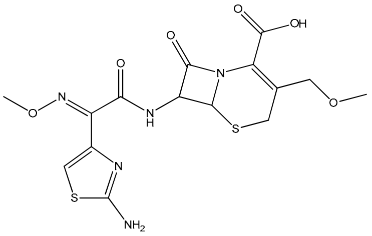

Cefpodoxime

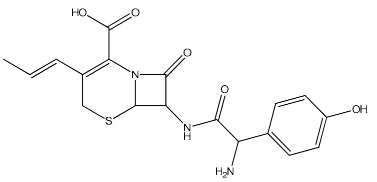

Cefprozil

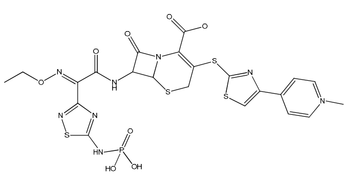

Ceftaroline fosamil

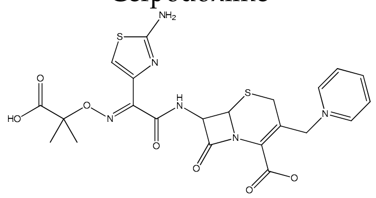

Ceftazidime

Figure S2. *Cont.*

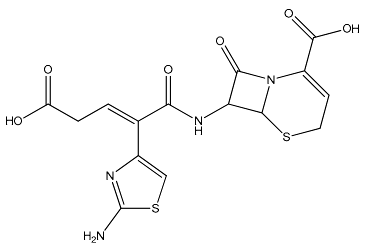

Ceftibuten

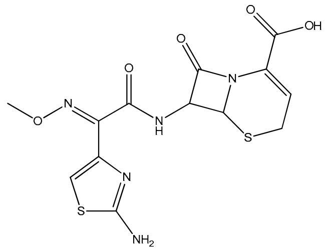

Ceftizoxime

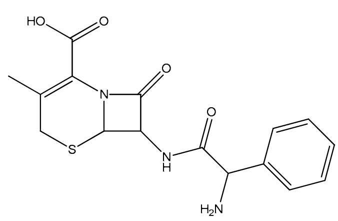

Cephalexin

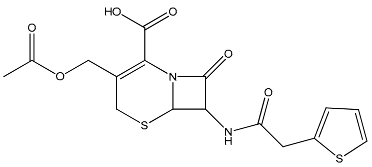

Cephalothin

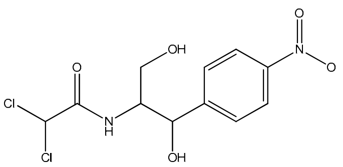

Chloramphenicol

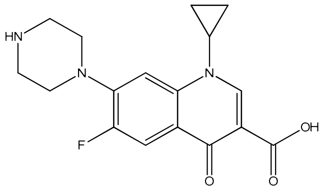

Ciprofloxacin

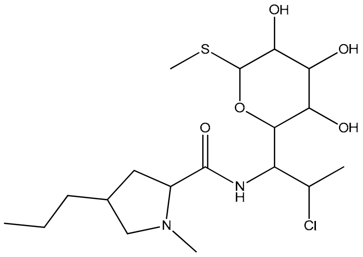

Clindamycin

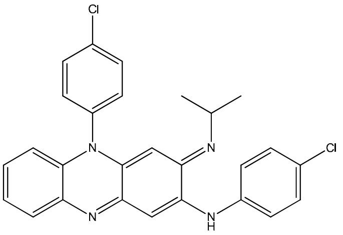

Clofazimine

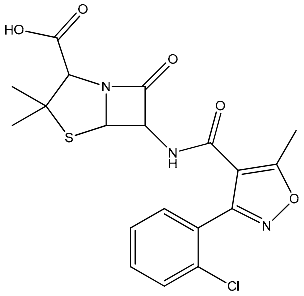

Cloxacillin

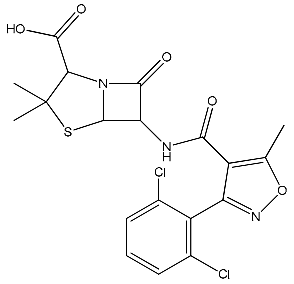

Dicloxacillin

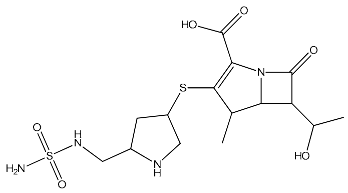

Doripenem

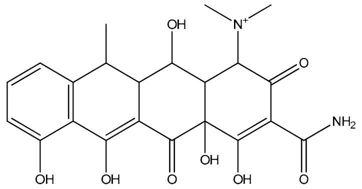

Doxycycline

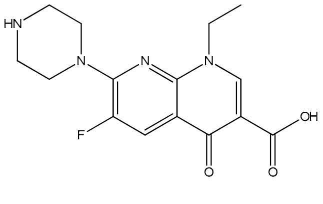

Enoxacin

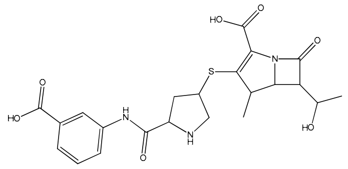

Ertapenem

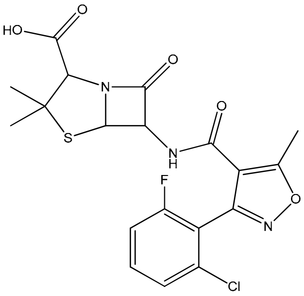

Flucloxacillin

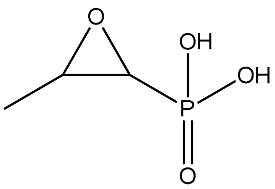

Fosfomycin

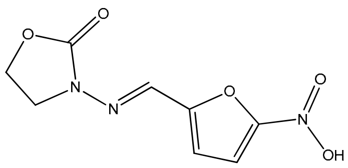

Furazolidone

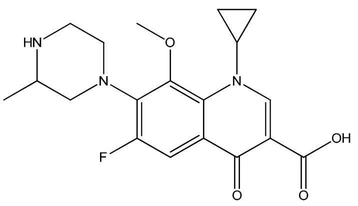

Gatifloxacin

Figure S2. *Cont.*

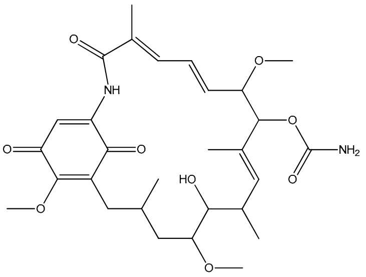
Geldanamycin

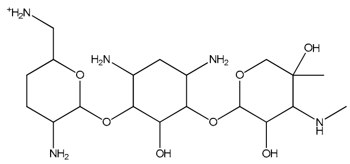
Gentamicin C

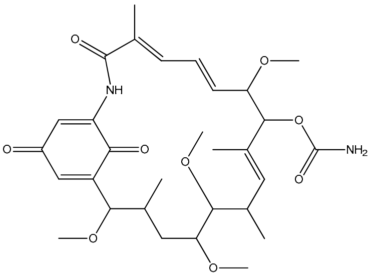
Herbimycin

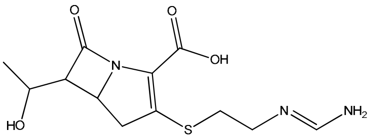
Imipenem

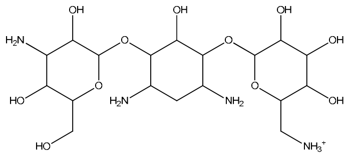
Kanamycin

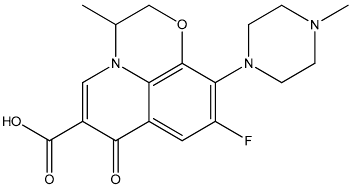
Levofloxacin

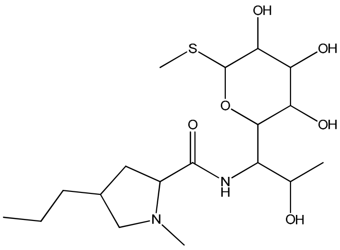
Lincomycin

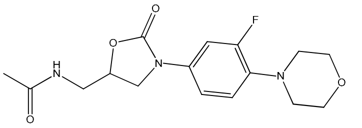
Linezolid

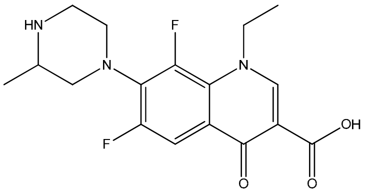
Lomefloxacin

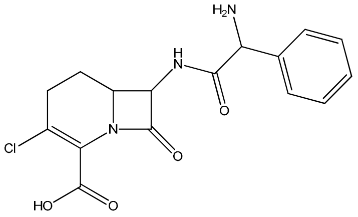
Loracarbef

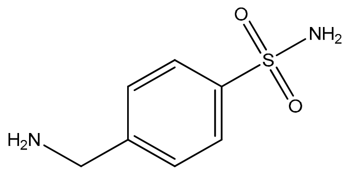
Mafenide

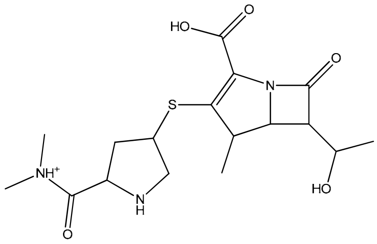
Meropenem

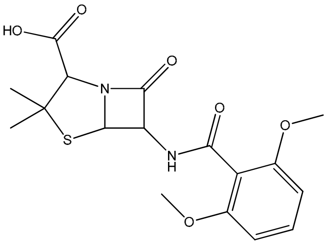
Methicillin

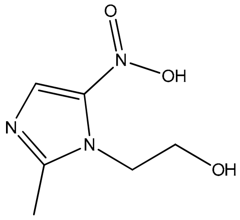
Metronidazole

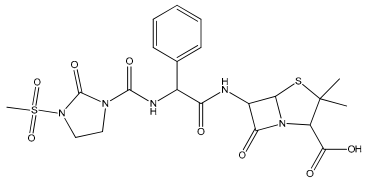
Mezlocillin

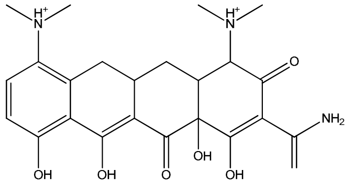
Minocycline

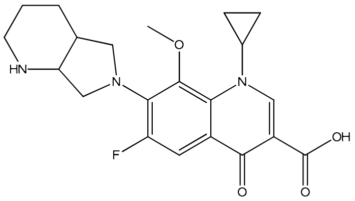
Moxifloxacin

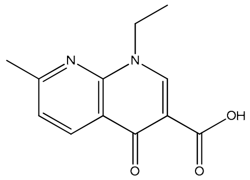
Nalidixic acid

Figure S2. *Cont.*

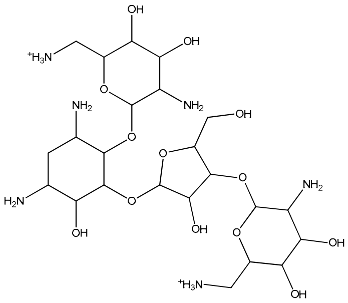

Neomycin

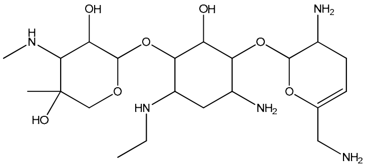

Netilmicin

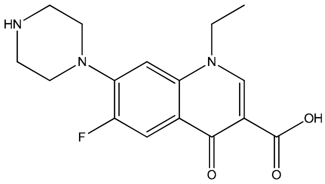

Norfloxacin

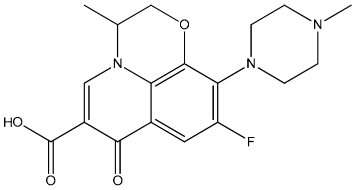

Ofloxacin

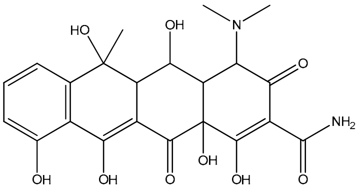

Oxytetracycline

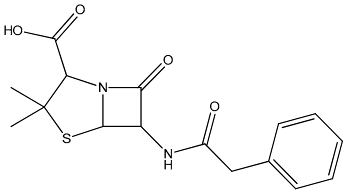

Penicillin G

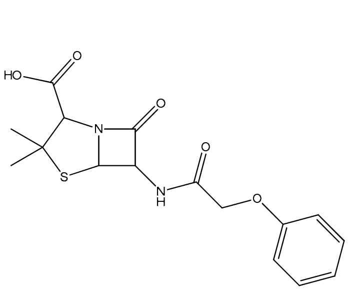

Penicillin V

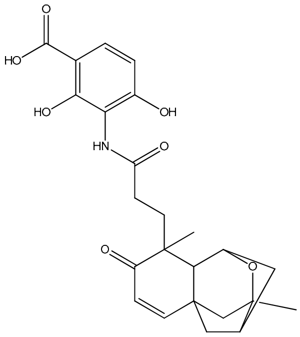

Platensimycin

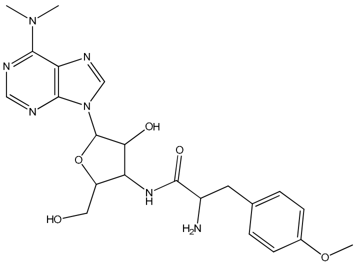

Puromycin

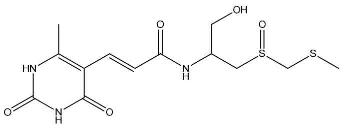

Sparsomycin

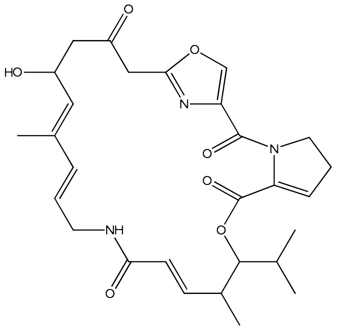

Streptogramin A

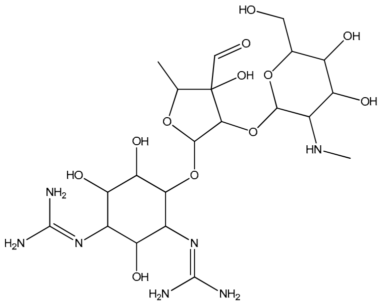

Streptomycin

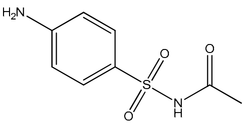

Sulfacetamide

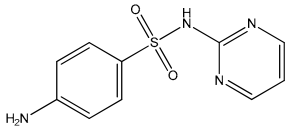

Sulfadiazine

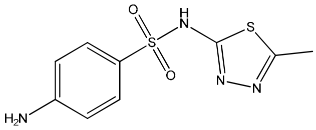

Sulfamethizole

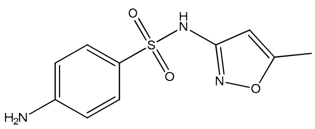

Sulfamethoxazole

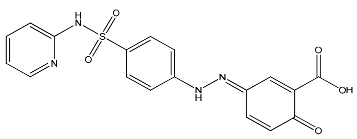

Sulfasalazine

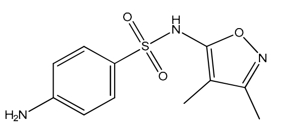

Sulfisoxazole

Figure S2. *Cont.*

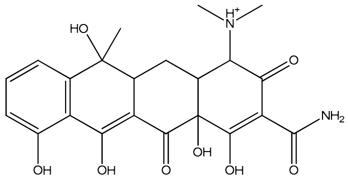

Tetracycline

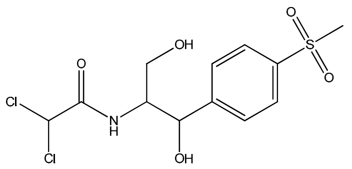

Thiamphenicol

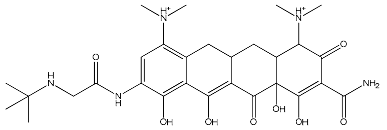

Tigecycline

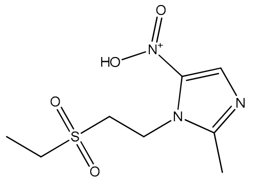

Tinidazole

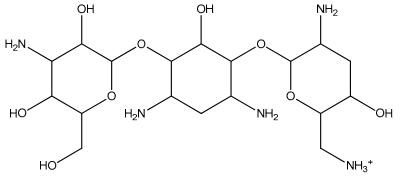

Tobramycin

Figure S2. Antibiotic ligands examined in this work.
